# An exploratory study of the relevance of therapy format and therapist’ training in individual cognitive behavioral therapy for psychosis

**DOI:** 10.3389/fpsyt.2025.1527549

**Published:** 2025-07-02

**Authors:** Oda Skancke Gjerdalen, Manuela Zucknick, Ingvild Tvedt Westlie, Helen Bull, Stig Evensen, Erik Falkum, Marit Grande, Roger Hagen, Kristin Lie Romm, Lene Hunnicke Jensen, Petter Andreas Ringen, Olivia Schjøtt-Pedersen, Nasrettin Sönmez, Torill Ueland, Tiril Østefjells, Jan Ivar Røssberg, June Ullevoldsæter Lystad

**Affiliations:** ^1^ Division of Mental Health and Addiction, Oslo University Hospital, Oslo, Norway; ^2^ Oslo Centre for Biostatistics and Epidemiology, Institute of Basic Medical Sciences, University of Oslo, Oslo, Norway; ^3^ Oslo Centre for Biostatistics and Epidemiology, Research Support Services, Oslo University Hospital, Oslo, Norway; ^4^ Faculty of Health Sciences, Oslo Metropolitan University, Oslo, Norway; ^5^ Institute of Clinical Medicine, University of Oslo, Oslo, Norway; ^6^ Independent Researcher, Sandvika, Norway; ^7^ Department of Psychology, Norwegian University of Science and Technology, Trondheim, Norway; ^8^ Research Institute, Modum Bad, Vikersund, Norway; ^9^ Department of Psychology, University of Oslo, Oslo, Norway; ^10^ Division of Mental Health and Addiction, Vestre Viken Hospital Trust, Drammen, Norway; ^11^ Department of Adult psychiatry, Diakonhjemmet Hospital, Oslo, Norway; ^12^ Department for Specialised Inpatient Treatment, Akershus University Hospital, Lørenskog, Norway

**Keywords:** cognitive behavioral therapy, psychosis, therapy format, therapist training, vocational rehabilitation

## Abstract

**Introduction:**

Cognitive Behavioral Therapy for psychosis (CBTp) is an evidence-based intervention that can be delivered in various formats, including as part of vocational rehabilitation. However, due to scarcity of resources, CBTp is currently accessible to only a minority of individuals with psychosis. This secondary analysis aims to explore potential differences in clinical outcomes between distinct CBTp formats and to examine whether therapist training influences treatment effect. Exploring these aspects is of importance, as they may influence the scalability and accessibility of CBTp in routine care.

**Material and methods:**

Data in this study is sourced from two independent projects; *KATOslo* and *JUMP*, comprising a total of 200 participants with broad schizophrenia-spectrum disorders. The current study compares CBTp delivered as either symptom-focused individual therapy (KATOslo) or as an add-on to a vocational rehabilitation (VR) program (JUMP), with two reference groups; VR combined with cognitive remediation (JUMP) and treatment as usual (KATOslo). Using a series of mixed effects models for repeated measurements, we examined differences between the groups in terms of general functioning and psychiatric symptom severity. Emphasis was placed on potential differences between the two groups receiving CBTp, considering both average differences across assessment points and trajectories over time.

**Results:**

In line with expectations, all groups demonstrated overall improvements in functioning and symptom levels. After adjusting for relevant confounders, no statistically significant differences were found between the two groups receiving CBTp following treatment initiation.

**Conclusions:**

These findings suggest that CBTp may be effective across different delivery formats and levels of therapist training in terms of similar, positive clinical outcomes for this patient group. This has potential implications for service design and broader implementation of CBTp in real-world settings.

## 1 Introduction

Cognitive Behavioral Therapy for psychosis (CBTp) is a well-established, evidence-based treatment, currently recommended in several international treatment guidelines for psychosis (e.g. 1). However, more recent meta-analyses have revealed only small to modest effects on symptoms and functioning when compared to treatment as usual (TAU) or other less sophisticated therapeutic approaches (2, 3).

CBTp – and CBT in general – can be considered as a family of complex interventions comprising various therapeutic components (4). Traditionally, CBTp is delivered in one-to-one sessions in the therapist’s office, focusing on identifying distressing thought- and behavioral patterns and promoting change through guided dialogue and between-session tasks (5). While in-session behavioral techniques and real-time exposure are emphasized, they often represent a limited portion of standard CBTp. However, CBTp can also be provided in other contexts and formats, such as in combination with vocational rehabilitation (VR) (6). Work-focused CBTp offers a systematic approach that integrates individual CBTp with job support. In such settings, CBTp may focus more explicitly on supporting individuals in managing mental health challenges in the workplace, emphasizing behavioral activation in naturalistic environments (i.e. an occupational context) (7, 8).

Supported employment schemes, such as Individual Placement and Support (IPS) have proven effective in supporting individuals with schizophrenia attain and sustain employment (9). These approaches have gained momentum as mental health interventions, given the potential benefits of employment, including improved quality of life and reduced symptoms of depression and anxiety (10, 11). Cognitive and metacognitive impairment is a prominent feature of psychotic disorders (12, 13). Studies have shown that enhancing VR with cognitive interventions, such as elements from CBTp or cognitive remediation, may further improve vocational functioning for this and other diagnostic groups (7, 14) and has been proposed as a possible path to maximize functional (occupational) outcome (15).

Despite clinical recommendations, CBTp remains inaccessible to many patients with psychotic disorders. In 2018, it was reported that only 26% of patients with psychosis in England and Wales were offered CBT (16). Barriers include limited dissemination within the health services (17), resource demands (18) and a shortage of psychologists and psychiatrists formally trained in CBTp. (19). It is therefore relevant to explore whether CBTp can be effectively delivered by professionals with varying levels of formal training with outcomes comparable to those achieved by highly trained specialists. Findings vary, but a systematic review found that therapists’ interpersonal functioning and skills had the strongest evidence of directly affecting treatment outcomes (20). Studies on CBT have found that neither therapist experience (21), nor other therapist characteristics significantly influenced patient outcome (22). Furthermore, some research suggests that therapists’ educational background and profession may not yield significant differences in outcome when providing CBT (23). However, a therapist’s adherence to the treatment manual is a factor that may be of particular importance in CBT and other manual-based therapies (24).

Since the various components of CBTp may be weighted differently depending on delivery format, it is important to explore how variations in settings and therapist background influence outcomes. This also aligns with the ongoing debate about CBTp’s effectiveness relative to other psychotherapeutic approaches (25). Examining the format in which CBTp is delivered could help identify these aspects. In this study, “CBTp format” refers to whether therapy is delivered in a traditional office-based setting or integrated into a work-oriented context.

This present study adopts a pragmatic approach by comparing secondary data from two independent CBTp trials. While the primary aims of the original studies and the CBTp formats differed, they shared similar assessment points and overlapping outcome measures and included comparable patient populations (see **Table 1**, below). The main aim of the present study was to explore the relevance of therapy format when delivering CBTp to individuals with broad schizophrenia-spectrum disorders. A secondary analysis was conducted to compare selected outcome measures from CBTp delivered in two different formats. One CBTp intervention was delivered by experienced CBT therapists with formal CBT training in a traditional, individually symptom focused context. The other CBTp intervention was delivered as an add-on to a VR program by employment specialists with less extensive CBT training. We examined whether these two CBTp formats differed in terms of symptom and functional outcomes over time. Additionally, we assessed whether differences in therapist training impacted outcomes. This exploratory study does not constitute an effect or non-inferiority trial but aims to generate insight into how delivery format and therapist background may influence CBTp effectiveness. To support interpretation and increase statistical power, we also included two reference groups from the original studies: one receiving TAU and another receiving VR combined with cognitive remediation. These groups were included to control for cohort effects and support modeling of time trajectories but are not the focus of the discussion. To our knowledge, this is the first direct comparison of traditional versus work-focused CBTp.

**Table 1:** Main components of the two CBTp interventions.

Component	JUMP	KATOslo
Therapy format	Add-on to a vocational rehabilitation program	Traditional therapy setting
Duration of the program	10 months	6 months
Duration of the CBTp intervention	6 months	6 months
Frequency of CBTp sessions	Once a week + additional homework assignment	Once a week
Average number of CBTp sessions and standard deviation (SD)	28,8 (SD = 12.4)	19,8 (SD = 6.6)
Personnel providing CBTp	Employment specialists (varied background, no doctors/psychologists)	Psychiatrists/psychologists/ occupational therapist
CBTp training	40 hours CBTp training + 8 hours education on psychosis	2-year CBT education by the Norwegian Association of Cognitive Therapy (128-192 hours)
Supervision	Weekly	Biweekly
Based on (literature)	Kingdon and Turkington (26)	Kingdon and Turkington (26) and Fennell (27)
Based on a treatment manual	Yes	Yes
Vocational rehabilitation program	Yes	No
Targeted focus area	Problems at the workplace	Emotional dysfunction

## 2 Materials and methods

### 2.1 Materials

This study draws on data from two independent projects: Cognitive behavioral therapy, Oslo (KATOslo) and the Job Management Program (JUMP). The studies are briefly described below. For full methodological, see Sönmez et al. (28) and Falkum et al. (6), respectively. In the current study, four groups are included; two groups receiving CBT in different settings/formats, and two reference groups: one group receiving TAU in KATOslo and one group receiving VR augmented with cognitive remediation (CR) in the JUMP study. Assessments were conducted at three time points: At baseline, post-intervention (6–10 months) and follow-up (15–24 months). The timing of assessments varied somewhat between the two studies and also between participants within each study. There was no calibration between assessors across the two studies, and no formal fidelity monitoring of therapy delivery was conducted.

#### 2.1.1 KATOslo

The KATOslo study is a randomized controlled trial (RCT) comparing CBTp with TAU in individuals with early psychosis. The main aims of the study were to examine whether CBTp compared to TAU would reduce depressive symptoms (primary outcome) and increase self-esteem (secondary outcome). Furthermore, the study aimed to examine whether CBTp reduced psychotic symptoms as measured with the PANSS (29) or increased general functioning compared to TAU. Fifty-two participants were included in the study and ongoing depressive symptoms were an inclusion criterion. Post-intervention assessments were conducted at 9 months on average (SD = 3 months) and follow-up assessments were carried out after 19 months (mean; SD = 4 months). The CBTp intervention was delivered by a dedicated CBT treatment team consisting of 2 clinical psychologists, 2 psychiatrists, and 1 occupational therapist. All therapists had completed a two-year educational program in CBT provided by The Norwegian Association of Cognitive Therapy. The program includes 29 days of lectures, training and supervision in CBT, and an exam at the end. In addition, all therapists attended monthly meetings to learn the specific CBTp manual and put it into practice. The meetings starting two years prior to the beginning of the study. Therapists received biweekly supervision during the intervention period. All participants continued to receive their ongoing usual treatment from their therapists/case managers in various psychiatric units in Oslo, Norway. The core components of TAU entailed ongoing medication, regular psychiatric review, and follow-ups by their case managers, often including some form of psychotherapy.

#### 2.1.2 JUMP

The JUMP study is a VR program for individuals with schizophrenia spectrum disorders, in which one intervention group received VR augmented with CBT, and the other group VR augmented with CR. Hundred and forty-eight individuals were included in the study. Participants were randomized to either CBT or CR based on their catchment area. The main outcome measure in the original study was employment status, including competitive work, work placement and sheltered work, and hours worked. Secondary outcomes were psychotic symptoms and cognitive functioning. Post-intervention assessments were conducted at approximately 9 months (mean; SD = 2 months) and follow-up assessments after 25 months (mean; SD = 3 months). The CR and CBT interventions were carried out by employment specialists with various educational backgrounds, but none were medical doctors/psychiatrists or psychologists. They received weekly supervision by experienced mental health professionals throughout the project. Forty hours of basic CBT training were offered to the employment specialists. They learned utilizing specific elements and techniques from CBT including cognitive restructuring, graded exposure, and homework, and training was geared at managing functional difficulties and counterproductive expectations related to situations at the workplace. Psychotic symptoms were not addressed per se, that is, only when interfering with occupational functioning. Employment specialists delivered CBT sessions once a week with additional homework assignments between sessions and received supervision during the intervention period. The sessions were tailored to the individual participant’s need and delivered both *in vivo* at the workplace, and at the employment specialists’ office. **Table 1** displays a summary of the core features of the two interventions concerning CBTp.

### 2.2 Measures

Demographic variables for both projects include age, gender, educational level, IQ as measured by WASI (30), and previous work experience. Primary diagnosis, daily dosage of antipsychotic medication (DDD), duration of illness (JUMP) and duration of untreated psychosis (KATOslo) were recorded in both projects. Alcohol and drug use were assessed by the self-report Alcohol Use Disorder Identification Test (AUDIT) (31) and the Drug Use Disorder Identification Test (DUDIT) (32), respectively. For diagnostic evaluation the M.I.N.I Plus (33) was used for the JUMP study, whereas the Structured Clinical Interview for DSM-IV Axis I disorders (SCID-I) (34) was used in the KATOslo study. Functioning was measured by the Global Assessment of functioning scale (GAF), split version (35) where general functioning and symptoms are rated from 0 (poorest) to 100 (best). Additional outcome measures were level of psychotic symptoms, as measured by Structural clinical interview – Positive and Negative Syndrome scale (SCI-PANSS) (29) where items are clinician rated from 1 (not present) to 7 (severely present). It is divided into subscales for positive, negative, and general symptoms (including disorganized/concrete, excited and depressive symptoms). Depression was evaluated by the Calgary Depression Scale for Schizophrenia (CDSS) (36), where clinicians rate nine items from 0 (absent) to 3 (severe). A cut-off score of ≥5 was used for inclusion in the KATOslo study. Social functioning was assessed using the Social Functioning scale (SFS) (37), which evaluates seven domains of social functioning. As employment status was the main outcome in the JUMP study, the SFS Employment subscale was included. We also examined self-esteem, as measured by Rosenberg Self-Esteem Scale (RSS) (38), as this was one of the main outcomes in the KATOslo study. This is a 10 items self-report measure, ranging from 0 (strongly disagree) to 3 (strongly agree), where higher scores indicate better self-esteem.

### 2.3 Participants

The demographic and clinical characteristics of the participants at baseline in the KATOslo and JUMP studies are displayed in **Table 2**.

**Table 2:** Demographic and clinical characteristics of the participants at baseline in the KATOslo and JUMP studies.

Variables	JUMP		KATOslo		P-value
	CBT JUMP (N=84)	CR JUMP (N=64)	CBT KATOslo (N=28)	TAU KATOslo (N=24)	
Age (years)					
Mean (SD)	33.0 (8.03)	32.3 (7.97)	29.4 (9.43)	27.7 (7.04)	0.008
Median [Min, Max]	32.5 [20.0, 57.0]	32.5 [20.0, 59.0]	25.0 [19.0, 51.0]	26.0 [18.0, 43.0]	
Gender					
Male	53 (63.1%)	50 (78.1%)	14 (50.0%)	15 (62.5%)	0.050
Female	31 (36.9%)	14 (21.9%)	14 (50.0%)	9 (37.5%)	
Education					
Primary school	26 (31.0%)	21 (32.8%)	3 (10.7%)	7 (29.2%)	0.310
High school	28 (33.3%)	22 (34.4%)	13 (46.4%)	5 (20.8%)	
Vocational education	8 (9.5%)	9 (14.1%)	2 (7.1%)	3 (12.5%)	
Collage/university	20 (23.8%)	12 (18.8%)	10 (35.7%)	9 (37.5%)	
Not completed primary school	2 (2.4%)	0 (0%)	0 (0%)	0 (0%)	
IQ (WASI)					
Mean (SD)	101 (14.2)	102 (13.5)	113 (11.2)	102 (12.5)	<0.001
Median [Min, Max]	104 [71.0, 133]	99.5 [71.0, 133]	116 [86.0, 129]	103 [79.0, 124]	
Missing	0 (0%)	0 (0%)	1 (3.6%)	3 (12.5%)	
Previous work experience (months)					
Mean (SD)	62.9 (68.5)	55.2 (63.9)	52.3 (58.7)	47.3 (59.8)	0.700
Median [Min, Max]	42.0 [0, 300]	24.0 [0, 240]	18.5 [1.00, 156]	18.0 [1.00, 168]	
Missing	0 (0%)	2 (3.1%)	16 (57.1%)	9 (37.5%)	
Antipsychotics DDD					
Mean (SD)	146 (259)	123 (208)	211 (333)	133 (183)	0.783
Median [Min, Max]	15.0 [0, 1200]	20.0 [0, 900]	20.0 [2.70, 1000]	20.0 [2.50, 600]	
Missing	0 (0%)	0 (0%)	5 (17.9%)	3 (12.5%)	
Diagnosis (psychosis)					
Schizophrenia	76 (90.5%)	55 (85.9%)	13 (46.4%)	14 (58.3%)	<0.001
Schizoaffective disorder	5 (6.0%)	6 (9.4%)	6 (21.4%)	3 (12.5%)	
Delusional disorder	2 (2.4%)	1 (1.6%)	2 (7.1%)	2 (8.3%)	
Psychotic disorder, INA	1 (1.2%)	2 (3.1%)	7 (25.0%)	5 (20.8%)	
AUDIT sum score					
Mean (SD)	4.55 (5.27)	4.63 (4.85)	6.76 (5.71)	5.46 (6.90)	0.215
Median [Min, Max]	2.00 [0, 27.0]	3.50 [0, 20.0]	6.00 [0, 19.0]	3.00 [0, 29.0]	
Missing	0 (0%)	0 (0%)	3 (10.7%)	0 (0%)	
DUDIT sum score					
Mean (SD)	1.89 (5.72)	1.66 (4.69)	4.36 (7.83)	3.38 (7.57)	0.045
Median [Min, Max]	0 [0, 39.0]	0 [0, 26.0]	0 [0, 33.0]	0 [0, 32.0]	
Missing	0 (0%)	0 (0%)	3 (10.7%)	0 (0%)	
SFS full scale score					
Mean (SD)	108 (6.94)	109 (6.92)	104 (10.2)	101 (9.45)	<0.001
Median [Min, Max]	108 [84.8, 122]	110 [89.9, 126]	104 [83.1, 125]	99.6 [83.6, 121]	
Missing	0 (0%)	0 (0%)	3 (10.7%)	2 (8.3%)	
SFS employment scale subscore					
Mean (SD)	105 (7.24)	106 (8.39)	106 (12.2)	109 (10.6)	0.1300
Median [Min, Max]	107 [95.0, 123]	107 [95.0, 123]	113 [81.5, 123]	113 [89.5, 123]	
Missing	0 (0%)	0 (0%)	3 (10.7%)	2 (8.3%)	
GAF-S score					
Mean (SD)	51.6 (9.54)	54.0 (11.2)	45.1 (13.2)	40.8 (8.89)	<0.001
Median [Min, Max]	51.0 [35.0, 81.0]	53.0 [29.0, 82.0]	40.5 [18.0, 76.0]	38.5 [30.0, 56.0]	
GAF-F score					
Mean (SD)	51.0 (8.59)	49.7 (10.3)	48.1 (11.6)	44.7 (9.72)	0.016
Median [Min, Max]	50.0 [30.0, 81.0]	48.0 [32.0, 78.0]	45.0 [22.0, 70.0]	42.5 [32.0, 65.0]	
RSS sum score (corrected)					
Mean (SD)	27.6 (5.57)	27.8 (5.28)	25.7 (5.08)	23.4 (4.15)	0.003
Median [Min, Max]	28.0 [15.0, 40.0]	27.0 [15.0, 40.0]	27.0 [14.0, 34.0]	24.0 [13.0, 30.0]	
Missing	2 (2.4%)	5 (7.8%)	0 (0%)	0 (0%)	
CDSS sum score					
Mean (SD)	3.51 (3.80)	4.26 (3.91)	7.39 (5.17)	8.33 (4.82)	<0.001
Median [Min, Max]	2.00 [0, 13.0]	3.00 [0, 16.0]	7.50 [0, 17.0]	8.50 [2.00, 17.0]	
Missing	1 (1.2%)	3 (4.7%)	0 (0%)	0 (0%)	
PANSS positive score					
Mean (SD)	12.5 (4.52)	14.0 (4.45)	13.5 (4.14)	14.2 (4.07)	0.090
Median [Min, Max]	11.0 [7.00, 22.0]	13.0 [7.00, 23.0]	13.5 [7.00, 23.0]	13.5 [7.00, 22.0]	
Missing	5 (6.0%)	1 (1.6%)	0 (0%)	0 (0%)	
PANSS negative score					
Mean (SD)	16.2 (5.74)	15.8 (5.57)	14.1 (4.92)	15.5 (4.46)	0.422
Median [Min, Max]	15.0 [7.00, 32.0]	15.0 [7.00, 29.0]	13.0 [7.00, 22.0]	15.0 [9.00, 24.0]	
Missing	5 (6.0%)	2 (3.1%)	0 (0%)	0 (0%)	
PANSS general score					
Mean (SD)	28.6 (8.62)	30.1 (7.58)	31.0 (7.24)	32.2 (5.45)	0.084
Median [Min, Max]	28.0 [16.0, 51.0]	29.5 [17.0, 51.0]	29.0 [20.0, 48.0]	33.0 [23.0, 42.0]	
Missing	5 (6.0%)	0 (0%)	0 (0%)	0 (0%)	
PANSS total score					
Mean (SD)	57.3 (16.4)	59.7 (14.1)	58.6 (13.8)	61.8 (10.9)	0.342
Median [Min, Max]	55.0 [30.0, 95.0]	59.0 [34.0, 97.0]	56.5 [36.0, 92.0]	65.0 [42.0, 81.0]	
Missing	5 (6.0%)	3 (4.7%)	0 (0%)	0 (0%)	

In KATOslo duration of untreated psychosis (DUP) in days was reported (CBT mean 122, SD 213, median 27 vs. TAU mean 193, SD 321, median 53), whereas duration of illness (DOI) in years was reported in the JUMP study (CBT mean 8.07, SD 6.83, median 7.00 vs CR mean 5.85, SD 5.47, median 4.00).

### 2.4 Statistical analysis

To compare groups, we estimated and compared the average (mean) trajectories over time of the selected outcome measures (PANSS total score, PANSS positive score, PANSS negative score, PANSS general score, GAF-S score, GAF-F score, SFS full scale score, SFS employment scale sub-score, RSS sum score corrected) between treatment groups. Given that test time varies somewhat between subjects, we first fitted mixed effects models allowing us to estimate the score for an average specific time point (baseline, post-intervention, and follow-up) with the lme4 R package version 1.1-35.1 (39). We allowed for non-linear time trajectories by using a flexible non-linear mixed effects model based on natural quadratic splines fitted with the splines R package version 4.3.2. We included the interaction effects between group and time, and adjusted for depression, as measured by CDSS, age at study inclusion and IQ as measured by WASI, as these were considered potential confounders. We checked the conditional R^2^ values (which take both the fixed and random effects into account) and marginal R^2^ values (which consider only the variance of the fixed effects), to evaluate the fit of the mixed effects models, for individual subjects as well as for the average trajectories (40) with the performance R package version 0.10.8 (41). The statistical significance of the individual fixed effects using Satterthwaite’s degrees of freedom method with the lmerTest package version 3.1-3 (42), and of the intervention group as a whole via likelihood-ratio tests. To compare only the two groups receiving CBT, the Wald test statistic was used for the relevant contrasts from the model. In addition, we explored the descriptive statistics of the participants. The demographic and clinical characteristics of the participants at baseline in the KATOslo and JUMP studies are compared using the Kruskal-Wallis tests for continuous variables and chi-squared tests for categorical variables. Due to clerical error, 4 inclusion dates in the KATOslo dataset could not be accurately identified. In these cases, the dates have been replaced by imputed values, in which the median across the observed values was used for imputation rather than the mean, due to skewed distributions. All statistical analyses were performed with the R language for statistical computing version 4.3.2 (43).

## 3 Results

Between-group differences at baseline in the KATOslo and JUMP studies were established on several demographic and clinical variables as shown in **Table 2** (above). Participants differed on age at inclusion, with KATOslo participants being slightly younger compared to the JUMP participants. The CBT group in the KATOslo study had higher IQ scores than the other groups. The KATOslo participants exhibited higher levels of ongoing depressive symptoms, as measured with the CDSS, which was anticipated since ongoing depressive symptoms were an inclusion criterion for entering the KATOslo study, but not for the JUMP study. There were also significantly different diagnostic distributions between the two samples. At baseline, the KATOslo participants displayed lower levels on both the GAF-F and GAF-S scales. Similarly, KATOslo participants had slightly lower scores on RSS sum score and SFS full scale. Consequently, we included age at inclusion, IQ as measured by WASI and CDSS scores as potential confounders in the subsequent analyses.

A series of mixed-effects model analyses were conducted to estimate the mean trajectories for each of the four intervention groups over time, based on the selected outcome measures. Overall, the four treatment groups exhibited distinct patterns for the outcome measures GAF-F, GAF-S, PANSS negative, and PANSS general scores. However, the groups showed similar patterns for the PANSS positive score, with no clear differences between the curves in any of the models. This, in turn, affects the PANSS total score, where all treatment groups show similar trajectories, except for the CR JUMP group, which exhibits a somewhat different pattern. For the GAF-F and GAF-S scores, the reference group TAU KATOslo generally shows a worse trajectory than the other three groups. In the GAF-S scores, both KATOslo groups show a downward trend between the post-intervention and follow-up timepoints, a trend not observed in the two JUMP groups. For the RSS sum score only the CR JUMP group has a significant main effect, with CBT JUMP reaching marginal significance (p=0.09). The CR JUMP and CBT KATOslo groups show significant interactions with time from baseline to post-test. However, none of these differences compared to the reference group are substantial relative to the observed between-subject variability. Overall, the group variable is not significant after adjusting for covariates, particularly differences in CDSS scores. The range of RSS scores is considerably wider in the JUMP cohort compared to the KATOslo cohort. In the final model for the SFS Full scale, only the CR JUMP group were statistically significantly different from the reference group, TAU KATOslo. It is important to note that the visible differences in mean score trajectories curves might be attributed to systematic variations in covariates between the groups (particularly the CDSS score) rather than inherent differences between the interventions/in the interventions themselves. For the SFS Employment scale, the JUMP groups are statistically significantly different from the reference group, TAU KATOslo, both in terms of main effects and the interaction with time from baseline to post-test.

**Figure 1:**
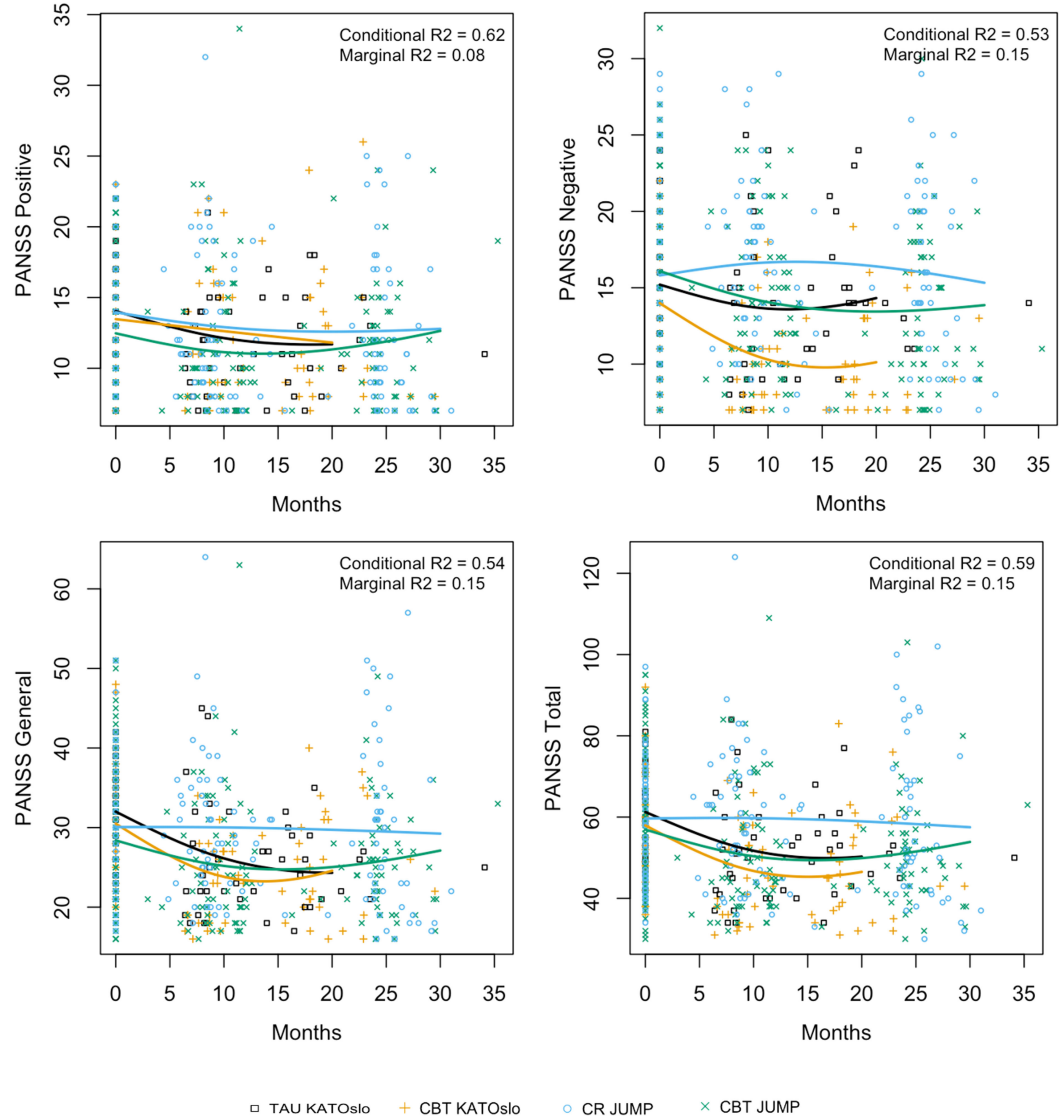
The estimated mean curves for each intervention group for the selected outcome measures PANSS positive, PANSS negative, PANSS general and PANSS total scores. The yellow curve (KATOslo) and the green curve (JUMP) depict the two CBT intervention groups. Due to shorter study length, the estimated curves from the KATOslo study are shorter than for the JUMP study.

**Figure 2:**
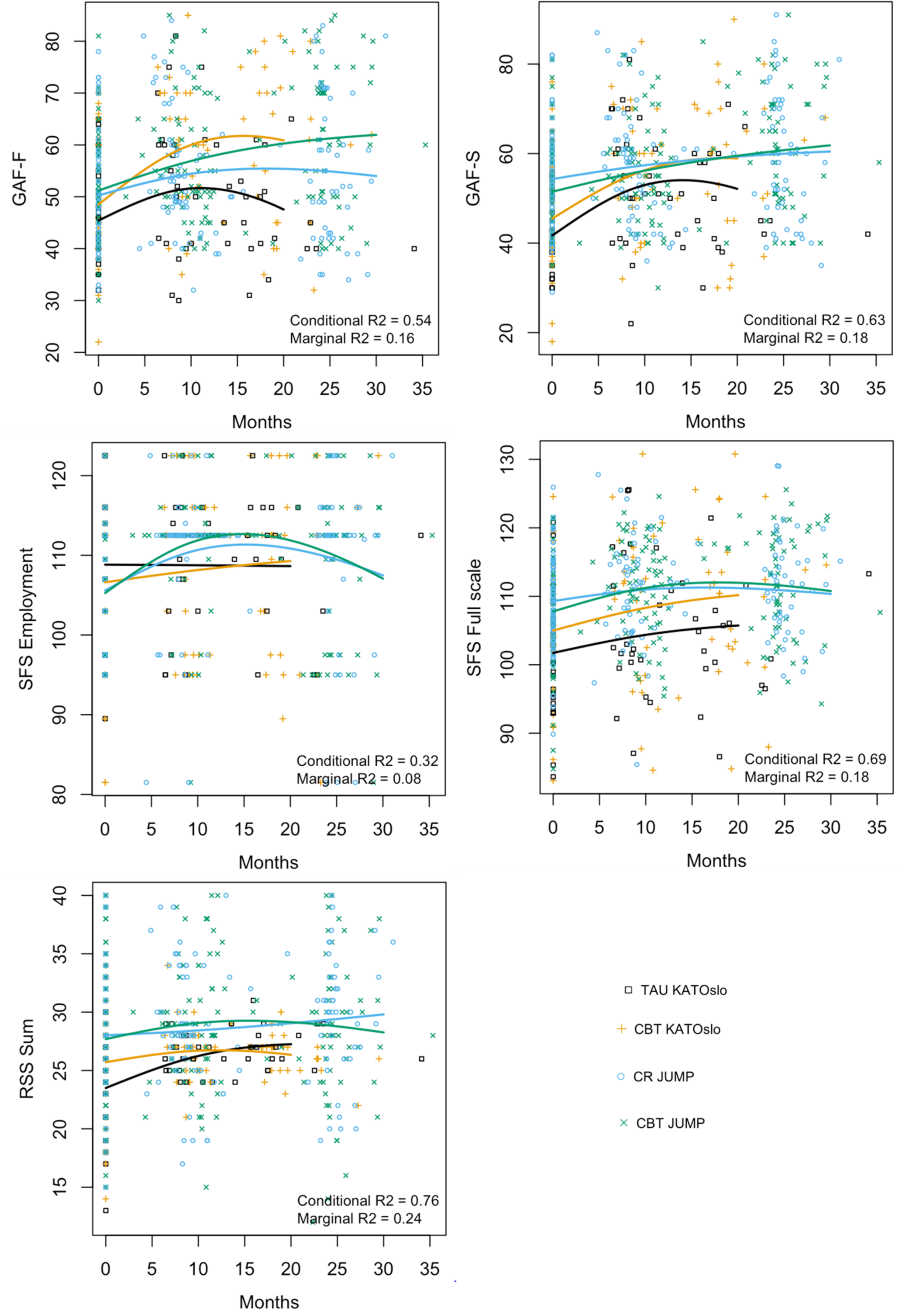
The estimated mean curves for each intervention group for the selected outcome measures GAF-F, GAF-S, SFS Employment scale, SFS Full scale and RSS. The yellow curve (KATOslo) and the green curve (JUMP) depict the two CBT intervention groups. Due to shorter study length, the estimated curves from the KATOslo study are shorter than for the JUMP study.

**Figures 1**, **2** below graphically present the results for GAF-F, GAF-S, the SFS full scale and employment subscale, the RSS sum score, as well as the PANSS scores for positive, negative, general psychopathology, and total symptoms. The graphs display the estimated mean curves for each of the intervention groups for the different outcomes. The yellow curve (KATOslo) and the green curve (JUMP) depict the two CBT intervention groups. The observed variance that is explained by the mixed effects models is reported by the conditional R^2^, which assesses both the fixed and random effects (the latter modelling the variability between study participants within each of the four intervention groups), and the marginal R^2^, which considers only the variance explained by the fixed effects, i.e. by the mean curves for each intervention group.

Detailed results for GAF-F, GAF-S, the SFS full scale and SFS employment subscale, the RSS sum score, as well as the PANSS positive, negative, general, and total scores are provided in Appendix 1. All groups are compared to the TAU group from the KATOslo study as reference group, with statistically significant results indicated by an asterisk (*).

Overall, after adjusting for the potential confounders CDSS, age at inclusion and IQ in the mixed effects models, no statistically significant differences were observed between the two different CBT groups, except for GAF-S at baseline. **Table 3** presents the results of the model-based comparisons between CBT KATOslo and CBT JUMP groups across the selected outcome measures. Statistically significant results according to two-sided tests at significance level 0.05 are indicated by an asterisk (*).

**Table 3:** Results comparing the two CBT groups for the selected outcome measures, based on the splines-based mixed-effects models, with adjustment for the potential confounding variables CDSS, age at inclusion and IQ (WASI).

Outcome measure	Estimate	Standard error	p-value
GAF-F			
** -** Difference at baseline	2.45	2.68	0.36
** -** Change to post-test^a^	-5.84	4.86	0.23
** -** Change to follow-up^b^	14.86	9.36	0.11
GAF-S			
** -** Difference at baseline	5.54	2.37	0.02*
** -** Change to post-test^a^	-9.62	5.05	0.06
** -** Change to follow-up^b^	8.04	8.93	0.37
SFS Full scale			
** -** Difference at baseline	1.12	1.68	0.51
** -** Change to post-test^a^	-1.73	3.52	0.62
** -** Change to follow -up^b^	-4.40	5.42	0.42
SFS Employment			
** -** Difference at baseline	-2.70	2.00	0.18
** -** Change to post-test^a^	6.99	4.37	0.11
** -** Change to follow-up^b^	-8.91	7.79	0.25
RSS Sum score			
** -** Difference at baseline	-0.03	1.04	0.98
** -** Change to post-test^a^	1.45	1.96	0.46
** -** Change to follow-up^b^	1.33	2.86	0.64
PANSS Positive			
** -** Difference at baseline	-0.70	1.01	0.49
** -** Change to post-test^a^	0.90	1.93	0.64
** -** Change to follow-up^b^	4.34	3.21	0.18
PANSS Negative			
** -** Difference at baseline	1.46	1.21	0.23
** -** Change to post-test^a^	2.15	2.31	0.35
** -** Change to follow-up^b^	-3.20	3.95	0.42
PANSS General			
** -** Difference at baseline	-0.81	1.69	0.63
** -** Change to post-test^a^	4.53	3.52	0.20
** -** Change to follow-up^b^	-6.64	6.07	0.28
PANSS Total			
** -** Difference at baseline	-0.04	3.28	0.99
** -** Change to post-test^a^	7.83	6.54	0.23
** -** Change to follow-up^b^	-4.96	10.97	0.65

**Figure 3:**
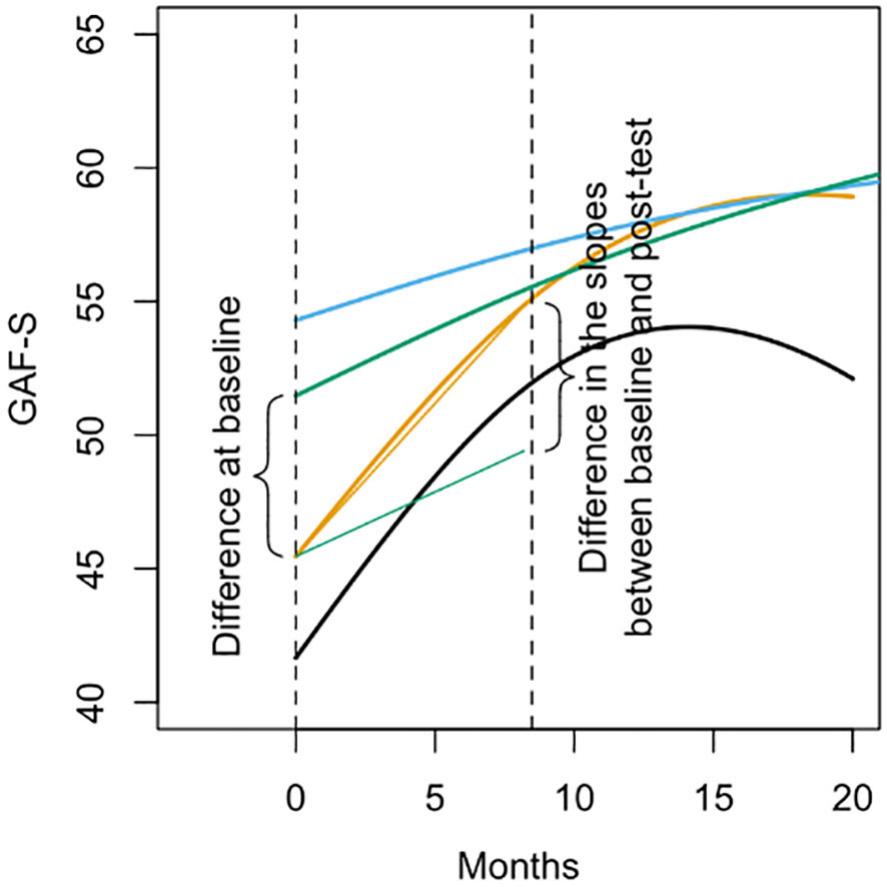
A visualization of the estimated parameters in **Table 3**.

**Figure 3** is a visualization of the estimated parameter in **Table 3**, using GAF-S as an example. The first parameter in **Table 3** represents the difference at baseline, as shown in the figure. The second parameter in **Table 3** represents the difference in the slopes between baseline and post-test, as shown in the figure. The third parameter in **Table 3** represents the difference in the slopes between post-test and follow-up. This is equivalent to the second parameter, but not included in the figure, as the difference is small and therefore difficult to visualize the figure.

## 4 Discussion

This secondary analysis examined whether the format in which CBT for psychosis (CBTp) is delivered, as well as the level of therapist training, influences clinical outcomes. Specifically, the goal was to compare traditional symptom-focused therapy delivered by therapists with formal CBTp training, to an integrated approach within a vocational rehabilitation program, provided by employment specialists with more limited CBTp training. In examining these distinct formats, we wanted to identify potential differences in participant outcomes and assess how varying levels of therapist CBT training might impact these effects.

The main finding of this study is that both CBTp groups demonstrated positive effects across all selected outcome measures. This aligns with expectations based on results from the original studies and is in line with previous literature highlighting the effects of CBTp (5, 44). Importantly, after adjusting for confounders, no statistically significant differences were observed between the two CBTp groups following the onset of treatment. This suggests that, despite differences in both intervention format and therapist training, the two CBTp approaches yielded comparable clinical benefits.

The two original studies had different primary aims (reduction of depressive symptoms in KATOslo vs. employment status in JUMP), which may have influenced the focus area of the interventions. However, CBTp is a goal-oriented intervention and focuses on the need of the individual. Thus, there may also have been different focus areas within the same study, not only between the studies. This is also underpinned by the considerable variability in the types of therapy offered under the single umbrella of CBTp (45, 46). There were also some differences in the demographic and clinical characteristics at baseline, e.g. age, IQ and level of depression (as measured by CDSS), but we have taken steps to control for these. Nevertheless, we acknowledge that these, and other differences in the study populations might affect the results observed in this study.

Prior research suggests that CBTp may be more effective in younger individuals and those with shorter illness duration (47), which is relevant given that participants in the JUMP study were, on average, older and had longer illness histories than those in KATOslo, where all participants were experiencing first-episode psychosis. Additionally, the average IQ in the KATOslo CBTp group was notably high (M = 113), exceeding what would typically be expected in this population. This has previously been identified as a potential source of systematic error in articles using these datasets and may partly reflect known issues of IQ overestimation in the Norwegian WAIS (48).

In the JUMP study, participants received a greater number of CBT sessions, as described in **Table 1**. However, this figure includes assignments and homework, meaning that the actual number of face-to-face sessions with a CBT therapist may have differed less than the raw numbers suggest. Nevertheless, the employment specialists in the JUMP study had more time and frequent contact with the participants, which may partly account for the observed effects. Due to the nature of their role, the employment specialists could also address difficulties in real-time, often accompanying participants in the workplace. In contrast, therapists in KATOslo primarily worked in traditional clinical settings, focusing on symptom discussions and cognitive strategies. For individuals with psychosis, applying such strategies outside of therapy can be challenging, particularly given the cognitive impairments often associated with psychotic disorders (13). Incorporating metacognitive assessments into CBTp may enhance engagement and treatment outcomes (12). When CBTp is delivered as part of a vocational rehabilitation (VR) program, it may offer additional benefits by integrating therapy into real-world contexts and addressing functional deficits alongside symptom reduction (49). The real-time, behavioral focus of JUMP may therefore have facilitated stronger generalization of skills and greater integration of therapeutic strategies into everyday life. Participants had opportunities to test new approaches, disconfirm negative beliefs, and challenge persistent delusional thinking *in vivo*, with direct support. Revisiting and engaging with the situation several days later, as seen in traditional CBTp, can be more difficult, requiring greater cognitive and emotional effort. This may underscore the particular importance of behavioral components in CBTp for schizophrenia-spectrum disorders.

However, it is essential to emphasize that this study was not designed as a non-inferiority trial. The absence of observed differences between the CBTp groups does not necessarily imply that the two interventions are equally effective or that no differences exist.

One could expect that participants receiving CBT from highly trained and more experienced therapist would do markedly better than the group receiving CBT from less trained personnel. However, research on therapist effect reports that considerable heterogeneity exists, and effects vary across studies (50), and one study suggests that the therapeutic relationship in CBTp is not affected by therapist’s experience (51). Our results suggest that the level of formal CBT training may be of limited importance, with regards to effectiveness, and that different amounts of CBT training can produce similar and positive outcomes. This is in line with a previous study which demonstrated that community psychiatric nurses could safely and effectively deliver CBT intervention to patients with schizophrenia (52). Furthermore, a recent study on delivery of psychotherapy showed non-inferiority of provider (specialist vs. non-specialist) and modality in treatment of perinatal depression (53). CBTp offered by therapists with two years of training in CBT, represents a costly intervention and a scarce resource. Enabling other professionals to offer CBTp may result in increased availability of the treatment, while not reducing the effectiveness of the intervention. This may play an important role for the implementing of CBTp to a larger proportion of patients with psychosis, as lack of trained personnel remains a considerable barrier (54). This is further highlighted in a review article on how mental health reforms may have significant implications for service design and equitable access, where persistent challenges such as resource shortages, regional disparities, and lack of continuity of care can critically shape how interventions like CBTp are adapted and delivered across varying service contexts and resource settings (55).

It also raises the question of adherence to manual. It may be speculated that employment specialists in the JUMP group, due to lack of experience and less training, adhered more to the specific manual than educated CBT therapists with more experience. Although experienced therapists with formal CBT training are expected to adhere to intervention protocols, it is possible that they exercise clinical judgement and adapt protocols to individual patient needs more frequently that therapists with less experience. Research on adherence to manual related to outcome in CBT is however inconsistent (56). Adherence may also be influenced by the complexity of cases, as CBTp sessions might have been interfered with other medical/psychiatric or psychosocial issues, time constrains, and patient preferences.

### 4.1 Strengths and limitations

The primary strength of this study lies in its exploration of factors that may influence the effectiveness of CBTp, specifically the therapy format and therapists’ CBT training. Reusing existing data, enable us to examine the impact of therapy format and therapists’ training on the delivery of CBTp to individuals with broad spectrum of schizophrenia disorders. This is a way of utilizing existing data, not originally intended by the individual studies, made possible when combining datasets. Data driven research fosters collaboration, reduces the need for duplicated data collection and makes existing data more accessible for broader research purposes, maximizing the value of each dataset. The two datasets used in this study had overlapping outcome measures, equal length of the CBT interventions (in months) and were performed at roughly the same time, in the same country. Thus, the data fit the secondary analysis design and research question (57). This study also utilizes a strong statistical method, using splines-based mixed effects models for modelling potential non-linear trajectories of the outcome measures over time and adjusting for potential confounding variables.

Several limitations should be considered when interpreting the results of this study. First, the original studies have different sample sizes, methodologies, and primary outcome measures (aims), which limit the direct comparability of the findings. There was a high degree of missing data regarding previous work experience for the KATOslo participants, but this was not part of the main analysis, and did therefore not influence results. Moreover, the studies included somewhat different populations, i.e. individuals with first episode psychosis in the KATOslo study vs. individuals with longer duration of illness in the JUMP study. Unfortunately, we lack DOI for KATOslo and DUP for JUMP, which makes direct comparison challenging. Observed diagnostic differences between the two samples is thus most likely due to JUMP participants being older, although non-significant, with a more established diagnosis, whereas KATOslo participants had experienced first episode psychosis, and may change the diagnosis to schizophrenia later in the course of illness. Furthermore, there is absence of blinding in outcome assessment in both original studies, no formal assessment of treatment fidelity and potential differences in supervision models across the cohorts/study populations. There are also unmeasured patient-level moderators, e.g. prior therapy exposure, that may influence effect of therapy (ref)?. Further research should aim to replicate and expand upon these findings, using larger and more diverse samples, standardized outcome measures and longitudinal design.

In the two original studies, interrater reliability was tested, but no interrater reliability tests have been performed between the two studies. This may be of significance, especially regarding GAF-scores, as concerns about its’ poor interrater reliability have been raised previously (58).

## 5 Conclusion

This exploratory study indicates that CBTp can be effective both when delivered by personnel with different levels of formal training and in different formats. Combining data in secondary analyses can be fruitful to further uncover the nuts and bolts of CBTp. There is however a need for confirmatory trials with more rigorous control over therapy delivery and therapist variables. Future research could profit from focusing on finding both patient and therapist characteristics, and delivery format that predict a positive response to specific treatment methods such as CBTp. Forthcoming studies should examine whether the behavioral component of CBTp might be of particular importance when delivering CBTp to this group.

## Data Availability

The original contributions presented in the study are included in the article/Supplementary Material. Further inquiries can be directed to the corresponding author.
