# Supplementary material for: An exploratory study of the relevance of therapy format and therapist’ training in individual cognitive behavioral therapy for psychosis
Source: Front Psychiatry. 2025 Jul 2;16:1527549. doi: 10.3389/fpsyt.2025.1527549 (PMC12263635; doi:10.3389/fpsyt.2025.1527549)

Appendix 1

Results for the different outcome measures when compared to the TAU group in the KATOslo study. The two parameters bs1 and bs2 define the basis matrix for the natural quadratic splines that allow non-linear time trajectories.

GAF-F


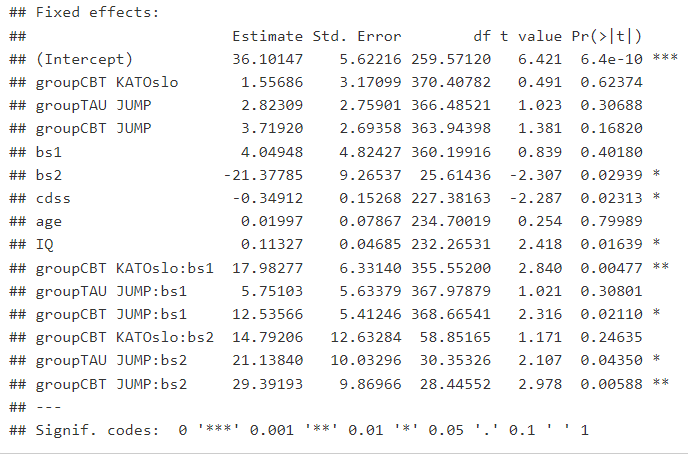


GAF-S


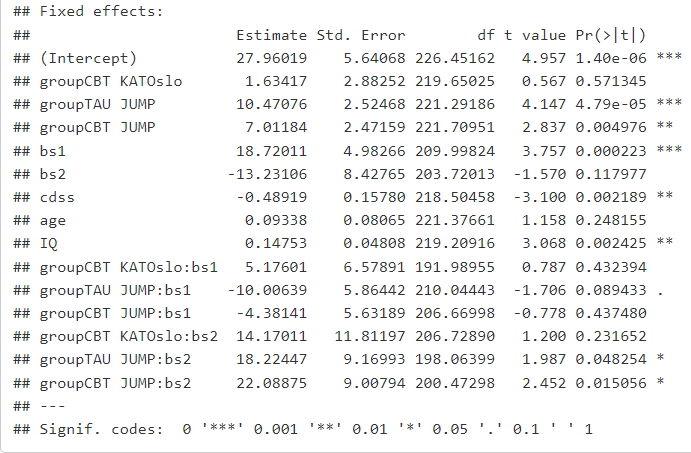


PANSS Positive


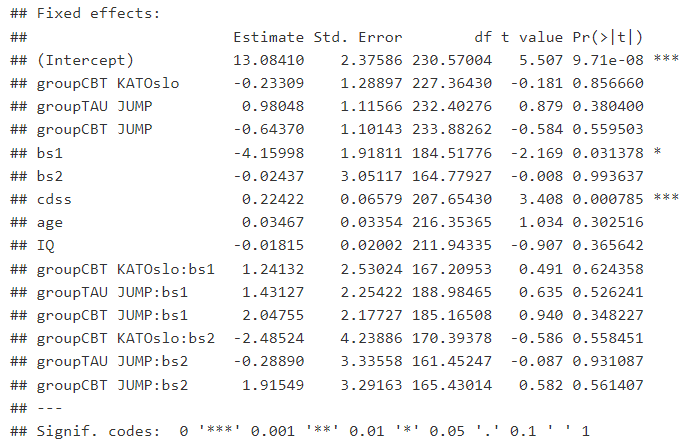


PANSS Negative


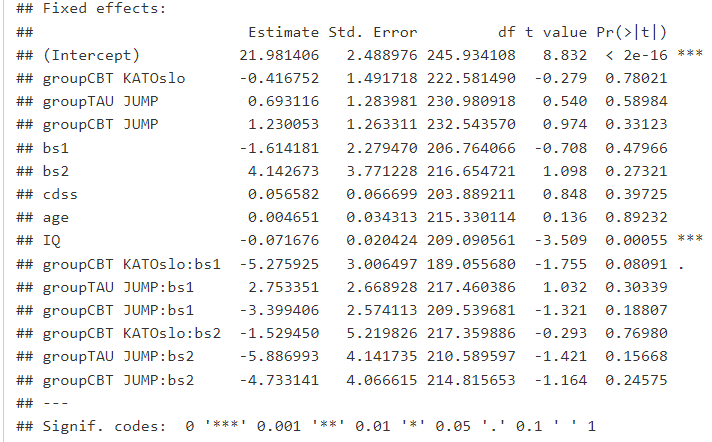


PANSS General


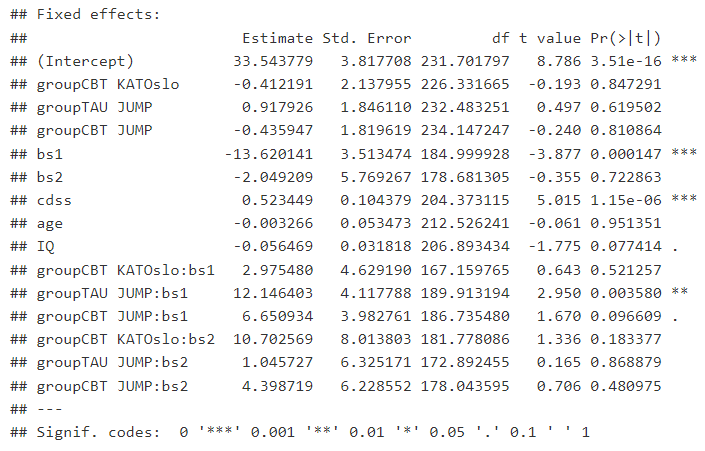


PANSS Total


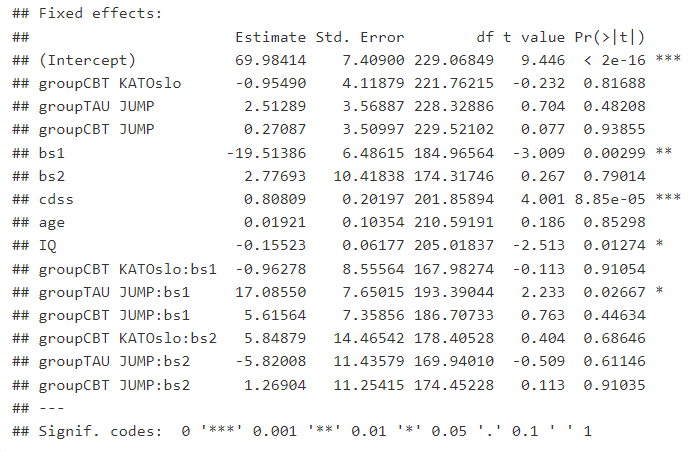


RSS Sum Score


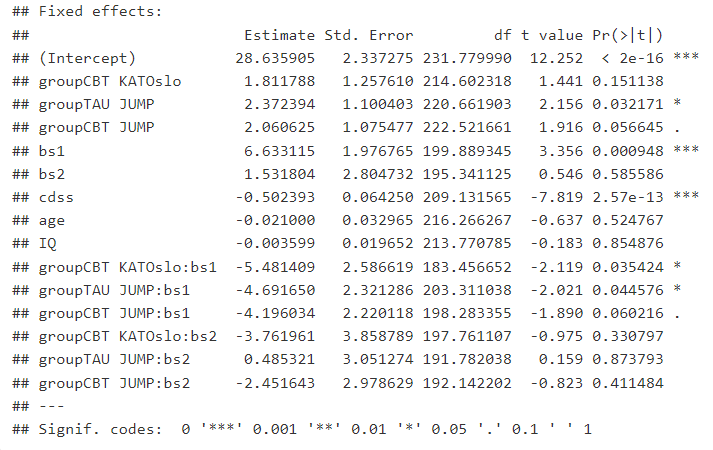


SFS Full scale:


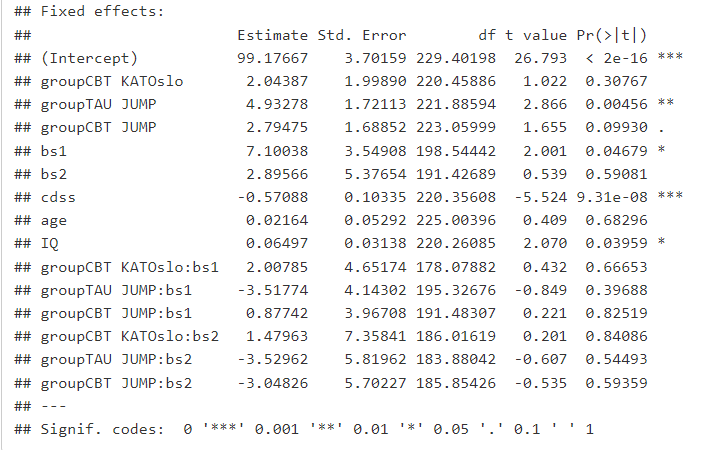


SFS Employment scale


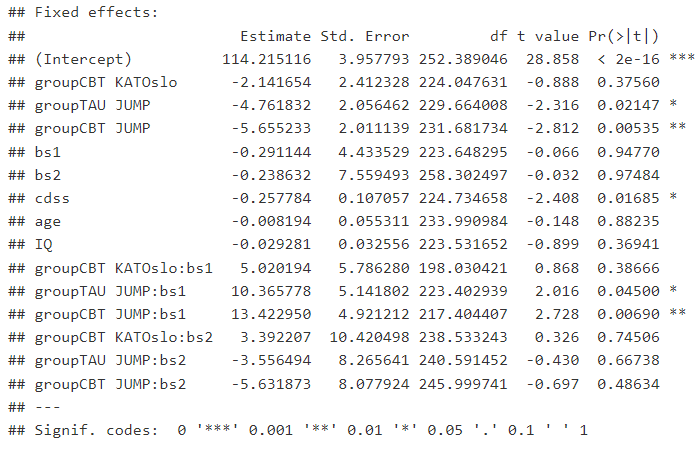

Supplement: Supplementary file 1 [file DataSheet1.docx]
